# The HIV Envelope but Not VSV Glycoprotein Is Capable of Mediating HIV Latent Infection of Resting CD4 T Cells

**DOI:** 10.1371/journal.ppat.1000633

**Published:** 2009-10-23

**Authors:** Dongyang Yu, Weifeng Wang, Alyson Yoder, Mark Spear, Yuntao Wu

**Affiliations:** Department of Molecular and Microbiology, George Mason University, Manassas, Virginia, United States of America; Harvard Medical School, United States of America

## Abstract

HIV fusion and entry into CD4 T cells are mediated by two receptors, CD4 and CXCR4. This receptor requirement can be abrogated by pseudotyping the virion with the vesicular stomatitis virus glycoprotein (VSV-G) that mediates viral entry through endocytosis. The VSV-G-pseudotyped HIV is highly infectious for transformed cells, although the virus circumvents the viral receptors and the actin cortex. In HIV infection, gp120 binding to the receptors also transduces signals. Recently, we demonstrated a unique requirement for CXCR4 signaling in HIV latent infection of blood resting CD4 T cells. Thus, we performed parallel studies in which the VSV-G-pseudotyped HIV was used to infect both transformed and resting T cells in the absence of coreceptor signaling. Our results indicate that in transformed T cells, the VSV-G-pseudotyping results in lower viral DNA synthesis but a higher rate of nuclear migration. However, in resting CD4 T cells, only the HIV envelope-mediated entry, but not the VSV-G-mediated endocytosis, can lead to viral DNA synthesis and nuclear migration. The viral particles entering through the endocytotic pathway were destroyed within 1–2 days. These results indicate that the VSV-G-mediated endocytotic pathway, although active in transformed cells, is defective and is not a pathway that can establish HIV latent infection of primary resting T cells. Our results highlight the importance of the genuine HIV envelope and its signaling capacity in the latent infection of blood resting T cells. These results also call for caution on the endocytotic entry model of HIV-1, and on data interpretation where the VSV-G-pseudotyped HIV was used for identifying HIV restriction factors in resting T cells.

## Author Summary

While receptor-mediated viral endocytosis or fusion with the cell membrane can be achieved through multiple surface molecules, the repetitious selection of two chemokine receptors, CCR5 or CXCR4, as the main HIV entry coreceptor implies an urgent viral need to exploit the chemotactic process in the immune system. Cytoskeletal rearrangement and cell migration are the primary consequences of chemotactic signaling. Nevertheless, previously published data demonstrated that depriving the virus of its signaling ability conferred higher infectivity through VSV-G-mediated endocytotic entry in transformed cells. We revisited the issue of chemokine coreceptor signaling and the role of cortical actin in HIV-1 latent infection of resting CD4 T cells, in which the virus can establish latency with a potential for productive replication upon T cell activation. Our results confirmed that only the genuine HIV-1 envelope protein, but not VSV-G, is capable of mediating latent infection of resting CD4 T cells. These findings highlight the importance of the HIV envelope and its signaling capacity in HIV infection of its natural target cells.

## Introduction

Binding of the HIV envelope to its receptors, CD4 and the chemokine coreceptor, CCR5 or CXCR4, triggers sequential fusion and entry events [1],[2],[3],[4]. Fusion is believed to occur directly at the plasma membrane [5],[6],[7],[8],[9], but fusion in endosomes has also been proposed recently [10]. It has been known that HIV can enter cells through endocytosis, but the virion particles entering through this pathway appear to be trapped and subsequently destroyed [11],[12]. This endosomal degradation can be rescued either by blocking the acidification of the endosomal compartments [11],[12] or by pseudotyping the HIV virion with the vesicular stomatitis virus glycoprotein (VSV-G) [13],[14]. The VSV-G-pseudotyped HIV escapes from endosomes and is highly infectious, giving the virus 20- to 130-fold higher infectivity [15],[16]. The ease of producing high-titer virus through VSV-G pseudotyping has made the method very popular for manufacturing viral stock used for gene delivery, drug screening, and the identification of cellular genes and factors involved in HIV replication [13],[17],[18],[19].

Nevertheless, the VSV-G-pseudotyped viruses are not identical to the genuine HIV particles. For example, the HIV Nef protein, a critical factor involved in viral pathogenesis [20], no longer plays an important role in the infection by the VSV-G-pseudotyped virus [15]. Nef has been known to enhance viral infectivity by a factor of 4 to 40 [21],[22]. This positive effect of Nef on viral infectivity appears to be at an early step post entry, such as uncoating or reverse transcription [23],[24],[25]. Nef itself does not directly affect reverse transcription, since Nef-defective virions display normal levels of endogenous reverse transcriptase activity [25]. It is likely that this early activity of Nef is connected to cortical actin in some way. For example, when cells were treated with actin inhibitors, the effect of Nef on viral replication was lost [26]. This is also consistent with the fact that the VSV-G-pseudotyped virus circumvents the cortical actin; thus, the impact of Nef on viral infectivity is forfeited most likely because of the lack of interaction with the actin cortex [15].

The VSV-G-pseudotyped HIV also does not engage CD4 and CCR5 or CXCR4, and is deprived of the ability to transduce signals through these receptors [27],[28],[29]. These intracellular signaling cascades, particularly those transduced from the chemokine coreceptors, have been suggested to be unnecessary for viral fusion, entry, or the subsequent steps of viral replication in transformed cell lines [30],[31],[32],[33],[34],[35],[36],[37],[38],[39]. However, recently, several reports have suggested a requirement for CD4 receptor signaling to mediate viral fusion and entry [40],[41],[42],[43]. We have also observed an absolute requirement for CXCR4 signaling in HIV-1 latent infection of resting CD4 T cells [44] and demonstrated that HIV-1 relies on viral envelope and the Gαi-dependent signaling from CXCR4 to activate a cellular actin-depolymerizing factor, cofilin, to increase the cortical actin dynamics for viral intracellular migration [44]. Given that the VSV-G-pseudotyped HIV infects cells in the absence of receptor signaling, we performed parallel studies in which the VSV-G-pseudotyped HIV was used to infect both transformed and resting CD4 T cells to understand possible alternative pathways that the VSV-G-pseudotyped HIV-1 may employ to establish latent infection of resting CD4 T cells. Surprisingly, the VSV-G-pseudotyped HIV-1 exhibited a highly diminished ability to initiate viral DNA synthesis and nuclear migration in resting T cells, which is in striking contrast to the high efficiency of VSV-G to mediate HIV infection of transformed cells. The viral particles entering through the endocytotic pathway were destroyed within 1–2 days in resting T cells. These results indicate that the VSV-G-mediated endocytotic pathway, although active in transformed T cells, is defective and not a pathway that can establish HIV latent infection of primary CD4 T cells. These results highlight the importance of the genuine HIV envelope and its signaling capacity in the latent infection of primary resting T cells.

## Results

### Characterization of the VSV-G-pseudotyped HIV replication in transformed T cells

**Figure 1:**
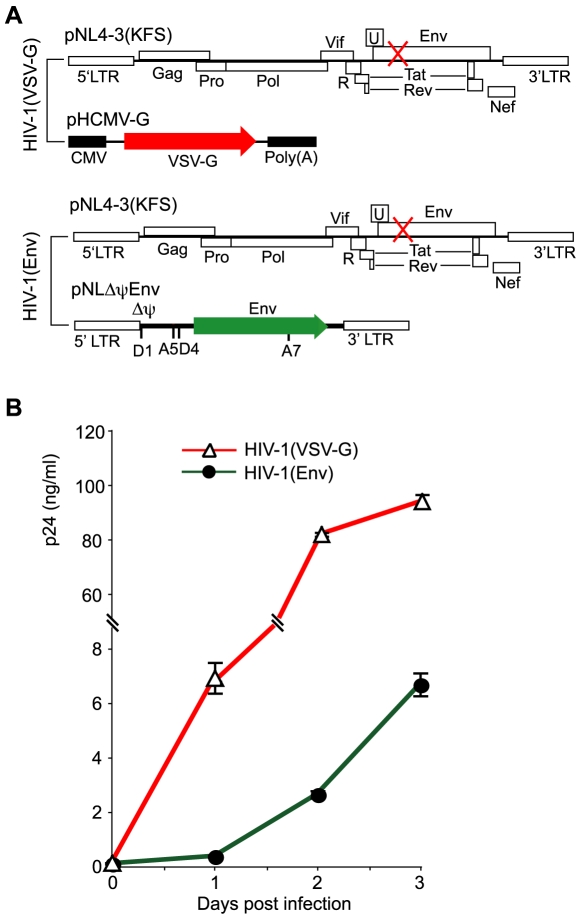
Replication of HIV-1 carrying either VSV-G or the HIV envelope in a transformed T cell, CEM-SS. (A) DNA constructs used to generate VSV-G-pseudotyped and HIV-1 Env-typed HIV-1. Viruses were generated by cotransfection and harvested at 48 hours. (B) An equal p24 level (260 ng) of HIV-1(VSV-G) or HIV-1(Env) was used to infect 1×10^6^ cells. Following infection for 2 hours, cell-free viruses were washed away and cells were continuously incubated for 3 days. Viral replication was monitored by p24 release.

We compared the infectivity of HIV-1 carrying either the HIV envelope (Wt) or the VSV glycoprotein (VSV-G). Both viruses were produced in parallel using the same cell culture and transfection conditions (Figure 1A). Following harvesting of viral particles, an equal p24 level of both viruses was used to infect a transformed T cell line, CEM-SS. Viral replication was monitored by p24 release. As shown in Figure 1B, we observed faster and stronger replication of the VSV-G- pseudotyped virus, which reached a level approximately 30 fold higher (at 48 hours) than the wild-type HIV-1. Our result was consistent with previous reports showing that the VSV-G-pseudotyped virus was 20 to 130 times more infectious than wild-type HIV-1 [15],[16]. It is likely that without the limitation of HIV receptors, much more productive viral entry may occur through the VSV-G-mediated endocytosis, resulting in a much higher level of viral replication. In addition, the faster replication kinetics of the VSV-G-pseudotyped virus is likely a result of faster entry and nuclear migration.

**Figure 2:**
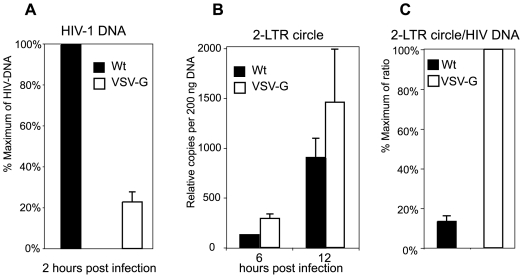
Comparison of viral DNA synthesis in CEM-SS cells infected with either HIV-1(VSV-G) or HIV-1_NL4-3_. Cells were infected with an equal TCID_50_ dose of HIV-1(VSV-G) or HIV-1_NL4-3_ (Wt) (1.27×10^5^ TCID_50/Rev-CEM_ per million cells). Following infection for 2 hours, cell-free viruses were washed away. Total cellular DNA was extracted from cells, and then amplified by real-time PCR to measure the synthesis of full-length HIV-1 DNA (2 hours post infection) (A) and 2-LTR circles at later time points (6 and 12 hours post infection) (B). The relative ratios of 2-LTR circles at 12 hours and HIV-1 DNA at 2 hours were plotted (C).

We also compared viral early processes after entry by following viral DNA synthesis and nuclear migration. We infected cells using an equal TCID_50_ dosage instead of an equal p24. Although more Wt particles were used (based on p24), infection with an equal TCID_50_ ensured that the productive viral processes such as viral DNA synthesis and nuclear migration would occur at comparable levels within the viral population in each case. The TCID_50_ of both viruses was measured on a Rev-dependent indicator cell, Rev-CEM, as previously described [45]. As shown in Figure 2, at 2 hours post infection, viral DNA synthesis was measured, and the VSV-G-pseudotyped HIV synthesized only approximately 20% of viral DNA in comparison with the wild-type virus (Figure 2A), probably either because fewer of the VSV-G-pseudotyped particles enter the cells or because these particles are less efficient at mediating viral DNA synthesis. We also followed viral nuclear migration at later time points using viral 2-LTR circles as a surrogate. As shown in Figure 2B, the VSV-G-pseudotyped virus produced slightly more 2-LTR circles than the wild-type HIV-1, and the relative ratio of 2-LTR circle to total viral DNA was approximately 7 times higher in the VSV-G-mediated infection (Figure 2C). These data suggest that in transformed T cells, the VSV-G-mediated endocytotic entry is much more efficient in delivering viral DNA into the nucleus. Even with a lower amount of viral DNA synthesized initially, a higher percentage of these DNA molecules entered the nucleus. On the other hand, in the wild-type infection, even with more viral DNA synthesis, a lower percentage of viral DNA molecules can enter the nucleus. These results are consistent with a model [46] in which the cortical actin plays an important role in viral reverse transcription [47], but the actin cortex also serves as a natural barrier for viral intracellular migration [44].

### Inability of the VSV-G-pseudotyped HIV-1 to establish latent infection of resting CD4 T cells

**Figure 3:**
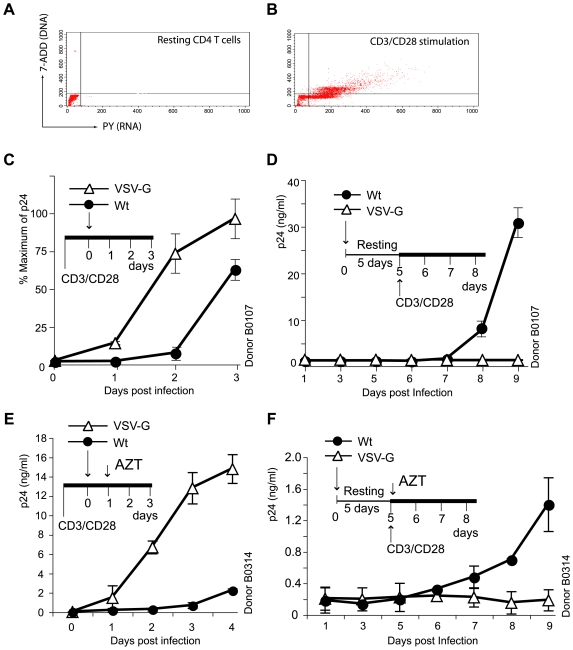
The HIV envelope but not VSV-G is capable of mediating latent infection of resting CD4 T cells. Resting CD4 T cells were purified from the peripheral blood by negative depletion. Cells were unstimulated (A) or activated with magnetic beads conjugated with antibodies against the human CD3 and CD28 receptors (two beads per cell) for 1 day (B), and then analyzed for cell cycle progression using 7-AAD, PY staining to confirm sufficient T cell activation following stimulation. (C) In CD3/CD28 pre-stimulated CD4 T cells, a higher than Wt level of viral replication was observed in cells infected with HIV-1(VSV-G). One million cells were infected with 25 ng (p24) of HIV-1_NL4-3_ (wt) or the VSV-G pseudotyped virus (VSV-G). (D) In resting CD4 T cells that were not pre-stimulated, only the Wt but not the VSV-G pseudotyped HIV-1 replicated following T cell activation at day 5. Cells were infected with 25 ng (p24) of both viruses, incubated for 5 days, and then activated with anti-CD3/CD28 beads. (E) and (F) were a repeat of (C) and (D) in another donor, with AZT (50 µM) added at day 1 and day 5 post infection, respectively, to limit viral replication to a single cycle.

In contrast to the VSV-G-mediated endocytotic entry, the HIV envelope-mediated fusion and entry requires specific interaction with CD4 and the chemokine coreceptor, CCR5 or CXCR4. These receptors not only mediate fusion but also transduce signals upon gp120 binding [27],[48],[49]. In particular, signals transduced from the chemokine coreceptor CXCR4 have recently been shown to be essential for HIV-1 latent infection of resting CD4 T cells [44]. Thus, we examined the ability of VSV-G-pseudotyped HIV-1 to establish latent infection of resting CD4 T cells in the absence of HIV coreceptor signaling. Resting CD4 T cells were purified from the peripheral blood of healthy donors by negative depletion (Figure 3A). Cells were rested overnight, and then infected with an equal p24 level of the VSV-G-pseudotyped HIV-1 or the wild-type HIV-1. Following infection, cells were washed and incubated for 5 days in the absence of T cell activation. During this incubation, productive viral replication does not occur. However, viral replication remains inducible upon T cell activation [44]. As a control, cells were also pre-activated for 1 day with antibody stimulation of the CD3/CD28 receptors (Figure 3B) and then identically infected. As shown in Figure 3C, in CD3/CD28 pre-activated T cells, productive viral replication occurred, and the VSV-G-pseudotyped viral replication was approximately 10-fold greater (48 h.p.i) than that of the wild-type virus. This is similar to the VSV-G-pseudotyped viral replication in transformed T cells (Figure 1). However, in latently infected resting CD4 T cells, when cells were activated at day 5 post infection, only the wild-type virus but not the VSV-G-pseudotyped HIV-1 was induced to replicate (Figure 3D). This was strikingly different from the 10 to 30-fold higher replication capacity of the VSV-G- pseudotyped virus in pre-activated and transformed T cells (Figure 1 and Figure 3C). These results were repeated using CD4 T cells from another donor with AZT added to limit viral replication to a single cycle (Figure 3E and 3F). Reproducibly, only the wild-type virus but not the VSV-G-pseudotyped virus was able to replicate following activation of resting T cells at day 5 (Figure 3F), even though the VSV-G-pseudotyped virus replicated to a 30-fold higher level (48h.p.i) in pre-activated T cells (Figure 3E).

### Inability of the VSV-G-pseudotyped HIV-1 to support viral DNA synthesis and nuclear migration in resting CD4 T cells

**Figure 4:**
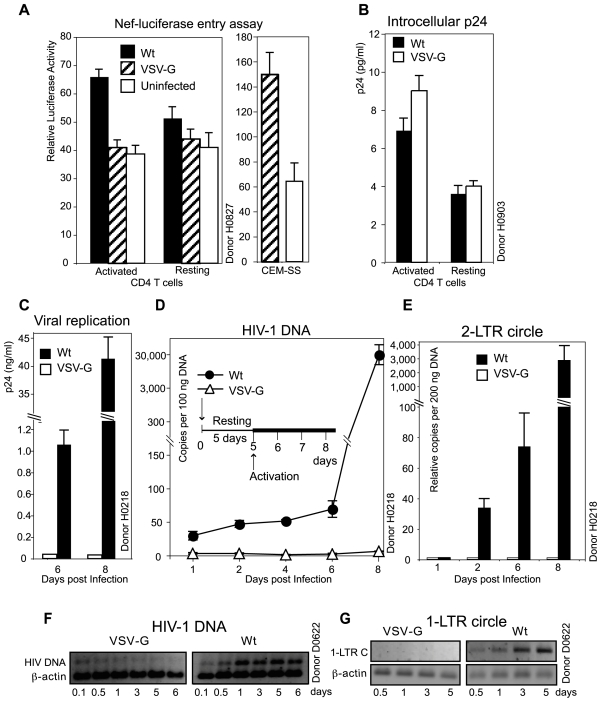
Measurement of viral entry and DNA synthesis following HIV-1(VSV-G) infection of resting CD4 T cells. (A) Resting or pre-activated (CD3/CD28 beads, two beads per cell) CD4 T cells (1×10^6^) were infected with 200 ng of Nef-luciferase-tagged HIV-1_NL4-3_ (Wt) or HIV-1(VSV-G) for 2 hours. Infected cells were washed three times and then used to measure luciferase activity in live cells. As a control, CEM-SS cells were identically infected with the Nef-luciferase-tagged HIV-1(VSV-G). Uninfected cells were identically treated and measured for luciferase activities. (B) Resting or pre-activated (overnight PHA plus IL-2 treatment) CD4 T cells (1×10^6^) were infected with 200 ng of HIV-1_NL4-3_ (Wt) or HIV-1(VSV-G) in 0.5 ml for 2 hours. Following infection, cells were treated with TrypLE (Invitrogen) for 2 minutes at 37°C and then washed an additional three times with medium. Cells were pelleted and subsequently lysed for p24 ELISA. (C to E) Resting CD4 T cells were infected with an equal TCID_50_ dose of HIV-1_NL4-3_ (Wt) or HIV-1(VSV-G) (2.53×10^5^ TCID_50/Rev-CEM_ per million cells). Following infection for 2 hours, cell-free viruses were washed away. Cells were cultured for 5 days and then activated with anti-CD3/CD28 beads. (C) A measurement of p24 release confirmed that only the Wt virus but not HIV-1(VSV-G) replicated following CD3/CD28 stimulation. (D, E) Total cellular DNA from infected cells was extracted at different time points, and then amplified with real-time PCR for HIV late DNA (D) or 2-LTR-circles (E). (F, G) is a repeat of (D, E) on another donor using an equal p24 level of Wt and HIV-1(VSV-G) to infect resting T cells. Total cellular DNA was extracted from infected cells at different time points and PCR-amplified for HIV late DNA (F) or 1-LTR circles (G), along with the β-actin pseudogene as a control.

We followed the steps for viral infection of resting T cells. Using a sensitive Nef-luciferase-based entry assay [50], we detected Wt viral entry into both resting and activated T cells (Figure 4A), although the entry into resting T cells was significantly lower. However, we could not detect viral entry into both resting and activated T cells in the VSV-G-pseudotyped virus infection, although we detected the entry of the VSV-G-pseudotyped virus into CEM-SS cells using the identical infection condition (Figure 4A). Since the VSV-G-pseudotyped HIV-1 can replicate in activated T cells (Figure 4C and 4E), these results suggested that the Nef-luciferase-based entry assay may not have the sensitivity to measure the VSV-G-mediated entry in primary T cells, either resting or activated. Thus, we used an alternative method to detect viral entry by measuring intracellular p24 following infection. Cells were infected for 2 hours, trypsinized, washed, and then lysed for intracellular p24. As shown in Figure 4B, we observed a comparable level of intracellular p24 in resting T cells infected with the wild-type or the VSV-G-pseudotyped HIV-1. We also observed a higher level of intracellular p24 in HIV-1(VSV-G)-infected active T cells (Figure 4B). These results suggested that entry of virion particles was similar in resting T cells infected with the wild-type HIV-1 or HIV-1(VSV-G).

We then followed the course of viral DNA synthesis and nuclear migration in resting CD4 T cells after infection. Unstimulated resting CD4 T cells from another donor were infected with an equal TCID_50_ dose of the VSV-G-pseudotyped HIV-1 or the wild-type virus. After washing away free viruses at 2 hours post infection, cells were continuously incubated without activation for 5 days, and then activated at day 5 with CD3/CD28 stimulation to initiate viral replication (Figure 4C). When viral DNA synthesis was analyzed, we did not observe viral DNA synthesis above the initial background (Figure 4D) in cells infected with the VSV-G-pseudotyped virus at any time point post infection, whereas in cells infected with the wild-type virus, we observed the typical course of viral DNA synthesis in which viral DNA synthesis proceeds slowly and usually peaks at day 2, recedes at day 3, and then increases again following T cell activation [44]. When viral 2-LTR circles were measured, in cells infected with the VSV-G-pseudotyped virus, we also could not detect 2-LTR circles at any time point, even after CD3/CD28 stimulation at day 5, whereas in cells infected with the wild-type virus, 2-LTR circles were detected and the copy number increased with time (Figure 4E). We repeated these experiments using resting CD4 T cells from another donor. This time, resting cells were infected with an equal p24 level of both viruses. We observed similar results. As shown in Figure 4F, in cells infected with the VSV-G-pseudotyped virus, the initial viral DNA detected (0.1 to 0.5 day in Figure 4F) diminished with time, and no 1-LTR circles can be detected at any time point post infection, whereas in the wild-type infected cells, the syntheses of both viral DNA and 1-LTR circles were obvious (Figure 4F and 4G). Based on these results, we concluded that in resting CD4 T cells, only the HIV envelope-mediated entry but not the VSV-G-mediated endocytosis can lead to viral DNA synthesis and nuclear migration, which are a prerequisite for the establishment of HIV latent infection of resting CD4 T cells [44].

### Decay of the VSV-G-pseudotyped HIV-1 in resting CD4 T cells

**Figure 5:**
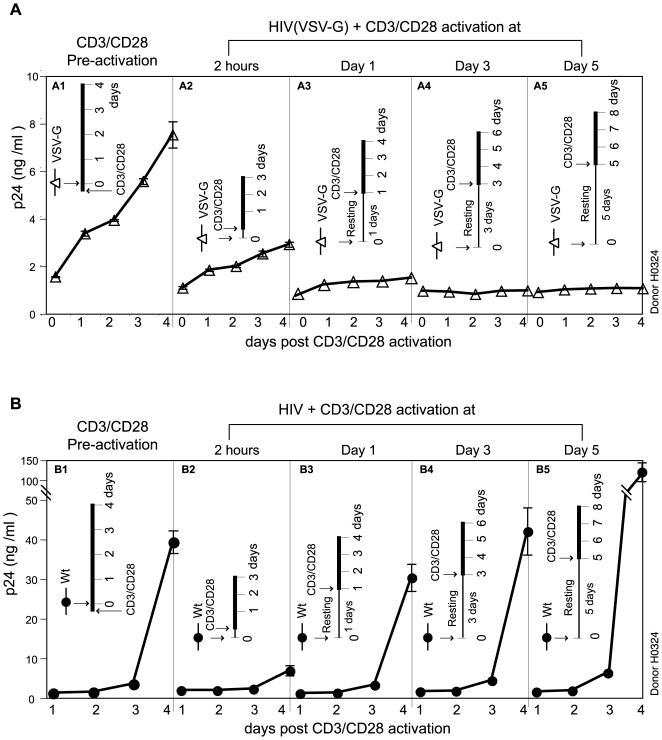
Decay of the VSV-G-pseudotyped HIV-1 in resting CD4 T cells. (A) Resting CD4 T cells were purified by negative depletion, rested overnight, and then pre-stimulated with anti-CD3/CD28 beads for 1 hour and infected with HIV-1(VSV-G) (552 ng p24 per million cells) (A1). Cells were also infected without pre-stimulation (A2 to A5) and then stimulated with anti-CD3/CD28 beads at 2 hours, day 1, day 3, or day 5 post infection to initiate viral replication. The p24 release was measured following anti-CD3/CD28 stimulation (marked as day “0” on the X-axis of each panel). (B) is a repeat of (A) in the same donor, using HIV-1_NL4-3_ (Wt) (552 ng p24 per million cells).

We also measured the decay kinetics of the VSV-G-pseudotyped HIV-1 in resting CD4 T cells. Unstimulated resting CD4 T cells were infected with an equal p24 level of both viruses. Following infection for 2 hours, cell-free viruses were washed away. Infected cells were then activated immediately or activated at day 1, 3, or 5 post infection. As a control, resting cells were also pre-activated with CD3/CD28 for 1 hour and then identically infected. As shown in Figure 5, in CD3/CD28 pre-activated CD4 T cells, both the VSV-G-pseudotyped HIV-1 and the wild-type virus replicated after infection (Figure 5A1 and 5B1). In resting T cells, when cells were activated immediately after infection and washing (2 hours post infection), viral replication was also initiated from both the VSV-G- pseudotyped HIV-1 and the wild-type virus (Figure 5A2 and 5B2). These results suggest that entry of the VSV-G-pseudotyped virus into resting T cells occurs, and viral replication can be rescued if cells are activated immediately. However, when resting cells were left unactivated, after 1 day, the replication of the VSV-G- pseudotyped virus following activation was greatly diminished (Figure 5A3), and no viral replication could be initiated after 3 days (Figure 5A4 and 5A5). This was in great contrast to the wild-type HIV infection of resting T cells, in which the capacity of HIV to replicate following activation increased with time (Figure 5B1 to 5B5). The highest viral replication occurred after 5 days of incubation. These data, in combination with the results in Figure 4, suggest that in resting CD4 T cells, the VSV-G-mediated endocytotic entry does not lead to a productive pathway, and the viral particles are trapped in cells and subsequently destroyed within 1–2 days. Our data are also consistent with a previous study demonstrating that the VSV-G-pseudotyped HIV-1 has a half-life of only 1–2 days in resting CD4 T cells [51]. The increasing ability of the wild-type HIV-1 to replicate following incubation has also been observed previously [52],[53],[54]. Although HIV does not directly replicate in resting CD4 T cells, the viral envelope-mediated entry establishes an active process that enhances the ability of HIV to replicate following T cell activation. This capacity has been attributed to the synthesis of Nef, which lowers the threshold required for the activation of resting CD4 T cells [52],[53],[54],[55],[56]. Certainly, our data confirmed these previous findings and further indicated that only the genuine HIV envelope protein but not the VSV-G can deliver the virus into the nucleus, where the subsequent action of Nef can occur.

### Differential inhibition of HIV-1 and the VSV-G-pseudotyped HIV-1 by dynasore

**Figure 6:**
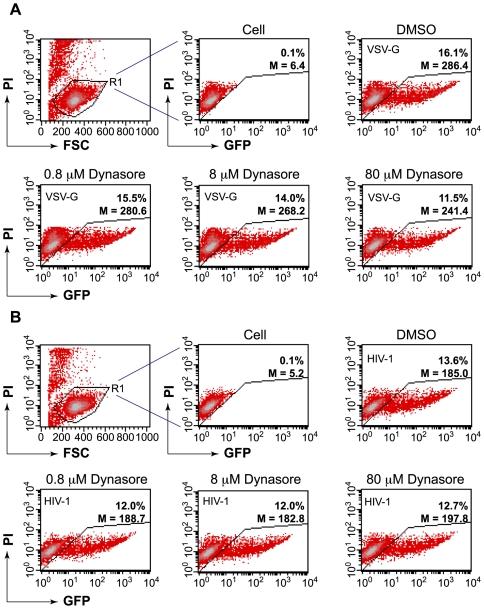
Different effects of dynasore on the replication of HIV-1(VSV-G) and Wt in a human T cell, Rev-CEM. (A) Rev-CEM, a Rev-dependent GFP indicator cell, was pretreated for 30 minutes with 80 µM, 8 µM, or 0.8 µM dynasore, respectively, or treated with 0.1% DMSO as a control. Cells were subsequently infected with HIV-1(VSV-G) in the presence of dynasore for 2 hours. Following infection, cells were washed three times with medium, and then cultured in the absence of dynasore. Viral replication was monitored by flow cytometry analysis of HIV-dependent GFP expression at 48 hours (20,000 cells analyzed per sample). Propidum iodide (PI) was added into the cell suspension prior to flow cytometry. Viable cells were gated (R1) based on low PI staining and cell size (FSC). GFP expression within the viable cell population (R1) was measured. Both the GFP percentage (%) and mean intensity (M) were shown. (B) is an identical experiment using HIV-1_NL4-3_ (Wt). For the 80 µM dynasore treatment, another three-independent infections with each virus were performed. The averages from the three-independent experiments are: 15.05%±0.21 (VSV-G), 10.11%±0.06 (VSV-G plus 80 µM dynasore), *p* = 0.0003; 2.94%±0.39 (Wt), 2.84%±0.30 (Wt plus 80 µM dynasore), *p* = 0.38.

In contrast to resting T cells, in transformed cell lines, the VSV-G-mediated entry is very efficient in mediating HIV infection. This fact has prompted a major argument that HIV may predominately fuse in the endosome rather than at the plasma membrane [10]. Microscopic imaging tracking the behaviors of the majority of MLV particles suggested that the HIV-1 envelope-pseudotyped virus entered cells predominantly through endocytosis [10]. Indeed, dynasore, a dynamin-dependent endosomal scission inhibitor, was shown to inhibit viral replication [10], supporting the model that the endosomal fusion is associated with a productive pathway. Nevertheless, this mode of entry is in conflict with numerous previous observations suggesting that genuine HIV envelope-associated endocytotic entry, although occurring at a significant scale, does not naturally lead to productive infection [5],[6],[7],[9],[11],[12]. For example, inhibition of the endosomal/lysosomal functionality can spare HIV from degradation and enhance viral replication [11],[12], demonstrating that the endosomal viruses are normally directed for degradation. In addition, the rate of CD4 or CCR5 endocytosis does not appear to affect viral entry or replication [6],[8],[9], supporting direct viral fusion at the plasma membrane. Nevertheless, the endocytosis entry as proposed [10] is an attractive alternative pathway. If proven biologically, it would require significant remodeling of the role of the cortical actin in viral entry and early post-entry steps. The involvement of the cortical actin in early endocytosis is largely limited to membrane scission of clathrin-coated pits [57]. This process does not involve direct contact between the cortical actin and the viral particles. If there is any viral contact with actin, it would be in the cytoplasm following endosomal fusion. This interaction may also affect reverse transcription and nuclear migration, but such effects would occur at different levels. The issue of entry is so critical in the understanding of the role of the cortical actin in HIV biology that we felt compelled to revisit some of the key biological evidence - in particular, the inhibition of HIV replication by the dynamin-dependent endosomal fusion inhibitor, dynasore. Dynamins are a group of fundamental proteins involved in multiple cellular processes such as vesicle transport, cytokinesis, organelle division and cell signaling (for a review, see [58]). To minimize possible cytotoxicity from prolonged inhibition of fundamental cellular proteins, we treated cells only briefly with dynasore during viral infection. Viruses that failed to enter were subsequently washed away along with the drug. We also used the Rev-dependent indicator cell, Rev-CEM [45], to measure dynasore inhibition, instead of simply using p24 ELISA, which by itself is not capable of distinguishing between HIV-specific inhibition and general drug cytotoxicity. Additional advantages of using Rev-CEM are its high specificity and the ability to distinguish subpopulations of cells by flow cytometry so that non-specific cytotoxicity can be excluded [59]. As shown in Figure 6A, we first tested dynasore in the inhibition of the VSV-G-pseudotyped HIV-1 replication and observed dosage-dependent inhibition of viral replication. At 80 µM, dynasore moderately decreased the GFP+ population from 16.1% to 11.5%; at 8 µM, dynasore also slightly decreased the GFP+ population; at 0.8 µM, dynasore minimally affected viral infection. However, when dynasore was used on identically treated cells that were infected with HIV-1, we did not observe similar dosage-dependent inhibition. Even at 80 µM, dynasore minimally affected HIV-1 infection (Figure 6B). These results demonstrate a clear distinction between the VSV-G-mediated endocytotic entry and the HIV-1-envelope-mediated entry in mediating productive viral replication.

## Discussion

In this report, we demonstrated a fundamental difference between the HIV-1 envelope and VSV-G in mediating HIV-1 latent infection of primary resting CD4 T cells, namely that only the HIV-1 envelope but not VSV-G is capable of supporting HIV latent infection of resting T cells. The block to the VSV-G-pseudotyped virus in resting T cells was most obvious at post-entry steps such as viral DNA synthesis and nuclear migration. The virion particles trapped in cells were subsequently destroyed within 1–2 days in resting T cells. These results demonstrated the importance of the genuine HIV envelope in mediating latent infection of resting T cells.

Previously, we demonstrated a critical function of the HIV-1 envelope in mediating CXCR4 signaling and promoting the cortical actin dynamics necessary for HIV latent infection of resting T cells [44]. We also proposed a dual function of F-actin in which the actin cortex serves as an anchorage for reverse transcription and as a vehicle for the delivery of the preintegration complex across the cortical actin through actin treadmilling [44],[46]. At least four HIV proteins in the preintegration complex are known to interact with actin; the viral nuclear capsid [60],[61],[62],[63], the large subunit of the reverse transcriptase [64], the integrase [65], and Nef [66]. We have also shown that blocking actin polymerization with Jasplakinolid (120 nM) or Latrunculin A (2.5 µM) inhibits viral DNA synthesis or HIV latent infection. Conversely, triggering actin polymerization through cofilin shRNA knockdown enhances viral DNA synthesis [44]. These previous results and other studies [47] are consistent with the findings in this study, in which the VSV-G-mediated entry that bypasses the cortical actin led to less viral DNA synthesis in transformed cells (Figure 2). The VSV-G-pseudotyping also resulted in a lack of the slow viral DNA synthesis that is normally seen in HIV-1 latent infection of resting T cells (Figure 4D and 4F). Viral nuclear DNA was also completely missing in the VSV-G-mediated entry in resting cells (Figure 4E and 4G). These results suggest that the VSV-G-pseudotyped particles may be delivered to a different cytoplasmic location and enter the nucleus by a different route, one that is normally highly effective in transformed or metabolically active cells but defective in resting T cells.

Our results are consistent with a recent independent study demonstrating that only the CXCR4-tropic HIV-1 envelope but not VSV-G can support lentiviral vectors to deliver genes into resting CD4 T cells [67]. In this study, Agosto and co-authors also found that viral DNA synthesis was greatly diminished in resting CD4 T cells infected with the VSV-G-pseudotyped lentiviral particles. Nevertheless, the limitation on viral infection was specifically attributed to the lack of viral entry and fusion in the VSV-G-mediated infection of resting T cells [67]. Our results suggested that the restriction was likely at an unknown post-entry step such as endosomal fusion, uncoating, or reverse transcription. The discrepancy in conclusions arises from different explanations of the data acquired from entry and fusion assays. Both Agosto and co-authors [67] and we observed an absolute lack of entry signals in HIV-1(VSV-G)-infected resting T cells, using two different entry assays. However, these assays, particularly the BlaM-Vpr-based fusion assay [68] may not be appropriate for the measurement of VSV-G-mediated fusion in resting T cells. It is possible that if the VSV-G-pseudotyped virus is trapped in a compartment, or is going through a degradation process with a half-life of only 1 day [51], the BlaM substrate which takes about 12–18 hours to load may not be able to access or sufficiently react with the enzyme. Given this lack of mechanistic clarity of how these enzyme-tagged particles are delivered through VSV-G in resting T cells, we did not feel confident that conclusions can be drawn based on a fusion assay. Thus, we drew our conclusions relying on multiple results. Firstly, we detected a comparable intracellular p24 level in resting T cells infected with Wt or HIV-1(VSV-G) (Figure 4B). Secondly, the VSV-G-pseudotyped virus can be partially rescued if resting T cells were activated within 1 day of infection, indicating some viral entry into the cells (Figure 5A2). Thirdly, low levels of viral DNA were also detected at early time (2 hours, Figure 4F), indicating again that there were some levels of entry. Given that the VSV-G-pseudotyped viruses are 20 to 130-fold more infectious than the wild-type HIV-1, these initial viral activities should give rise to a measurable level of viral replication, but they did not.

The failure of the VSV-G-mediated entry to establish latent infection of resting T cells is not currently understood. It is possible that the cellular environment in resting T cells may not permit viral fusion in endosomes. Alternatively, successful endosomal fusion may occur, but the quick delivery of viral particles into the cytosol may be detrimental [69], likely due to the possible restrictive environment of resting cells [17],[70] or a lack of cytosolic factors for uncoating [71] DNA synthesis, or nuclear localization. Our attempts to rescue the VSV-G-pseudotyped virus by changing the intracellular PH were not successful (data not shown). Pre-stimulation of the CD4 and CXCR4 receptors with gp120 or antibodies also could not rescue the VSV-G-pseudotyped virus in resting T cells (data not shown), although these pre-stimulations enhanced the wild-type HIV replication several fold following T cell activation [44]. These results are consistent with the fact that the positive benefits of viral receptor signaling are only associated with gp120-mediated entry but not with the VSV-G-mediated endocytosis that circumvents the cortical actin.

The high efficiency of VSV-G to mediate endosomal escape and HIV replication in transformed cells has led to the misconception that the VSV-G-pseudotyped HIV should be as effective as the wild-type HIV for latent infection of resting T cells [72],[73]. Several previous studies have also used the VSV-G-pseudotyped virus to identify restriction factors in resting T cells [17],[74]. Our results suggest that these data need to be interpreted cautiously. Apparently, the VSV-G-mediated entry does not experience the same intracellular environment as HIV does, and cannot lead to the establishment of latent infection in resting T cells. Thus, those previously identified cytoplasmic restriction factors may or may not directly affect HIV infection. Interestingly, a recent imaging study demonstrated a direct dependence of active viral nuclear migration on F-actin, since actin inhibitors diminished the nuclear concentration of the preintegration complex (PIC) (Dr. Thomas Hope, personal communication). This study raises the possibility that PIC may be associated with F-actin up to the nucleus [75],[76]. Given that viruses usually use F-actin for short-distance travel, and the cytoplasmic space between the cortical actin and the nucleus is relatively thin in T cells, it is possible that the cytosolic exposure of PIC in T cells is minimal.

## Materials and Methods

### Ethics statement

All protocols involving human subjects were reviewed and approved by the GMU IRB. Informed written consents from the human subjects were obtained in this study.

### Plasmids and DNA cloning

Plasmid pNL4-3 was kindly provided by Dr. Malcolm Martin [77]. The env mutant, pNL4-3(KFS), was kindly provided by Dr. Eric Freed [78]. pHCMV-G that expresses the vesicular stomatitis virus glycoprotein has been described previously [79]. pNLΔΨEnv was constructed by inserting the env gene of HIV-1_NL4-3_ into the lentiviral vector pNL-RRE-SA [80]. The packaging signal was further deleted by cutting with *Kas*I plus *BssH*II and re-ligating.

### Viruses and cells

HIV-1_NL4-3_ was generated by transfection of plasmid pNL4-3 into HEK293T cells using lipofectamine 2000 (Invitrogen) as described previously [80]. The VSV-G-pseudotyped virus, HIV-1(VSV-G), was produced by cotransfection of HEK393T cells (3×10^6^) with 10 µg of pHCMV-G and 10 µg of plasmid pNL4-3(KFS). The HIV-1 envelope-typed virus, HIV-1(Env), was produced by cotransfection of HEK293T cells with 10 µg of pNLΔΨEnv and 10 µg of pNL4-3(KFS). Viral supernatant was harvested at 48 hours post cotransfection, centrifuged for 15 minutes at 500×*g* to remove cellular debris, filtered through a 0.45 µm filter, treated with Benzonase (Novagen) (250 U/ml) at 37°C for 15 minutes, and then stored at -80°C. Levels of p24 in viral supernatant were measured using the Perkin Elmer Alliance p24 antigen ELISA Kit (Perkin Elmer). Plates were kinetically read using an ELx808 automatic microplate reader (Bio-Tek Instruments) at 630 nm. Viral titer (TCID_50_) was determined on the Rev-dependent GFP indicator cell, Rev-CEM [45],[81]. CEM-SS cells from Dr. Peter L. Nara [82] were obtained through the AIDS Research and Reference Reagent Program, Division of AIDS, NIAID, NIH. All cells were cultured in RPMI 1640 medium supplemented with 10% heat-inactivated fetal bovine serum (Invitrogen), penicillin (50 U/ml), and streptomycin (50 mg/ml).

### Isolation, culturing, and infection of resting CD4 T cells

Peripheral blood mononuclear cells (PBMC) were obtained from healthy donors at the Student Health Center, George Mason University (GMU), Fairfax, VA. Resting CD4 T cells were purified by two rounds of negative selection as previously described [54]. Purified cells were cultured in RPMI 1604 medium supplemented with 10% heat-inactivated fetal bovine serum (Invitrogen), penicillin (50 U/ml), and streptomycin (50 µg/ml) overnight before infection or treatment. For activation of resting CD4 T cells with PHA (3 µg/ml) (Sigma) plus IL-2 (100 U/ml) (Roche Applied Science), cells were cultured in the presence of these agents for 12 hours. For infection, CD4 T cells were incubated with the virus for 2 hours and then washed twice with medium to remove unbound virus. Infected cells were resuspended in fresh RPMI 1604 medium supplemented with 10% heat-inactivated fetal bovine serum at a density of 10^6^ per ml and incubated for 5 days without stimulation. Cells were activated at day 5 with anti-CD3/CD28 magnetic beads at 4 beads per cell. For the viral replication assay, 10% of infected cells were taken at days 1, 3, 5, 6, 7, 8, and 9 post infection. Cells were pelleted and the supernatant was saved for p24 ELISA.

### CD3/CD28 bead conjugation and stimulation of resting CD4 T cells

Monoclonal antibodies against human CD3 (clone UCHT1) and CD28 (clone CD28.2) were purchased from BD Pharmingen (BD Biosciences). For conjugation, antibodies were conjugated with 4×10^8^ Dynal beads (Invitrogen) for 30 minutes at room temperature. Free antibodies were washed away with PBS-0.5% BSA. The conjugated magnetic beads were resuspended in 1 ml of PBS-0.5% BSA. For stimulation of resting CD4 T cells, antibody-conjugated beads were washed twice and then added to cell culture and rocked for 5 minutes.

### Cell cycle analysis by 7-AAD and PY staining

Resting CD4 T cells or CD3/CD28-stimulated cells (10^6^) were used for the analysis. Before staining, magnetic beads were removed by incubating with DNase I releasing buffer as recommended by the manufacturer. Cells were suspended in 1 ml of 0.03% saponin in PBS and then incubated in 20 µM 7-amino-actinomycin D (Sigma) for 30 minutes at room temperature in the dark. Cells were kept on ice for at least 5 minutes, pyronin Y (Sigma) was added to a final concentration of 5 µM, and the cells were then incubated for 10 minutes on ice. Stained cells were directly analyzed by flow cytometry on a FACS (Becton Dickinson FACSCalibur).

### Production of HIV-1 and VSV-G-pseudotyped HIV-1 containing Nef-luciferase fusion protein for entry assay

Plasmid pCDNA3-Nef-Luc was kindly provided by Dr. Robert Davey [50]. Viruses containing Nef-luciferase was produced as described previously [50]. Briefly, 293T cells cultured in a 10 cm petri dish were cotransfected with 10 µg pNL4-3 plus 10 µg of pcDNA3-Nef-luc, or with 10 µg pNL4-3(KFS) plus 7.5 µg pcDNA3-Nef-luc plus 2.5 µg pHCMV-G, using lipofectamine 2000 (Invitrogen) as recommended by the manufacturer. Viruses were harvested at 48 hours post cotransfection and filtered through a 0.45 µM filter. For entry assays, cells (1×10^6^) were infected with 200 ng of Nef-luciferase containing viruses at 37°C for 2 hours, and then washed three times with medium. Cells were resuspended in 0.1 ml of luciferase assay buffer (Promega) and luciferase activity was measured in live cells using a GloMax-Multi Detection System (Promega).

### PCR and Real-time PCR

Total cellular DNA was purified using the Wizard SV Genomic DNA Purification System as recommended by the manufacturer (Promega). The detection of viral late DNA and 1-LTR-circles by PCR was performed as described previously [83]. Briefly, for viral late DNA, forward primer: 5′ GGTTAGACCAGATCTGAGCCTG 3′ and reverse primer: 5′ TTAATACCGACGCTCTCGCACC 3′ were used. PCR was carried out in 1×Ambion PCR buffer, 125 µM dNTP, 50 pmol each primer, 1 U SuperTaq Plus (Ambion) with 30 cycles at 94°C for 20 seconds, 68°C for 40 seconds. For detection of 1-LTR circle, primers LTR-nef2 (5′ TGGGTTTTCCAGTCACACCTCAG 3′) and LTR-gag (5′ GATTAACTGCGAATCGTTCTAGC 3′) were used. The reaction was carried out in 1×Ambion PCR buffer, 1.5 nM Mg^2+^, 125 µM dNTP, 50 pmol each primer, 1 U SuperTaq Plus (Ambion) with 35 cycles at 94°C for 20 seconds, 68°C for 90 seconds. Real-time PCR quantification of viral late DNA and 2-LTR circles was also performed as described previously [44],[84], using 300 nM primers and 200 nM probes. The DNA standard used for both late DNA and 2-LTR circle quantification was constructed using a plasmid containing a complete 2 LTR region (pLTR-2C); the plasmid was cloned by amplification of infected cells with 5′-TGGGTTTTCCAGTCACACCTCAG-3′ and 5′-GATTAACTGCGAATCGTTCTAGC-3′. Measurement was run in triplicate ranging from 1 to 10^6^ copies of pLTR-2C mixed with DNA from uninfected cells.

### Confocal microscopy

FITC-phalloidin staining of F-actin has been described previously [44]. Stained cells were imaged using a Zeiss Laser Scanning Microscope, LSM 510 META, with a 40 NA 1.3 or 60 NA 1.4 oil DIC Plan-Neofluar objective. Images were processed and analyzed by LSM 510 META software.

### Flow cytometry

Dynasore monohydrate (Sigma) was dissolved in DMSO. Following dynasore treatment, infection, and washing, cells were incubated for 48 hours, and then 500 µl cells were removed and stained with 2 µg/ml propidium iodide solution (Fluka) for 5 minutes at room temperature. Following incubation, cells were analyzed using the FACSCalibur (BD Biosciences). Data analysis was performed using CellQuest (BD Biosciences).
